# Nutrition as a missing link in One Health: from conceptual invisibility to strategic action

**DOI:** 10.3389/fnut.2026.1884842

**Published:** 2026-07-08

**Authors:** Júlio Belo Fernandes, Diogo Sousa-Catita, Jorge Fonseca

**Affiliations:** 1 Egas Moniz Center of Interdisciplinary Research (CiiEM), Egas Moniz School of Health and Science, Almada, Portugal; 2 Residências Montepio, Serviços de Saúde, Lisboa, Portugal

**Keywords:** food systems, health promotion, interprofessional collaboration, nutrition, One Health

## Abstract

The One Health approach recognizes the interdependence between human, animal, and ecosystem health and calls for integrated, system-oriented responses to contemporary health challenges. This viewpoint argues that One Health depends on the coordinated engagement of all health professionals, and that dietitians (also referred to as nutritionists, as the professional title varies across countries) play a particularly strategic role due to the systemic reach of their practice. Operating at the interface of food systems, dietary behaviors, population health, and environmental sustainability, dietitians are well-positioned to address upstream determinants of health across the life course. We highlight how nutrition practice advances One Health objectives, often without being explicitly recognized as One Health action by professionals themselves. We discuss key barriers to conceptual uptake and propose strategies to make nutrition's contribution visible, intentional, and strengthened through education, interprofessional collaboration, research, and evaluation frameworks. Making One Health explicit in nutrition practice is essential to translating the paradigm into sustainable action and addressing interconnected challenges of environmental degradation, health inequities, and long-term population well-being.

## Introduction

The One Health concept is grounded in the recognition that human, animal, and ecosystem health are deeply interconnected and mutually dependent (1). In a global context shaped by climate change, the emergence and re-emergence of zoonotic diseases, antimicrobial resistance, food insecurity, and persistent social inequalities, it has become increasingly clear that contemporary health challenges cannot be effectively addressed through isolated, discipline-specific approaches (2). Responding to this complexity requires the coordinated mobilization of all health professionals, working in integrated, collaborative, and systems-oriented ways (2, 3).

Within this framework, One Health constitutes a transversal paradigm across health professions, including physicians, nurses, dietitians, pharmacists, psychologists, physiotherapists, and others involved in addressing health determinants across the life course. Each profession contributes complementary competencies essential for translating conceptual principles into sustainable practice (4, 5).

Among these professions, nutrition occupies a significant position, especially when One Health is framed not only as a preventive approach but as a paradigm strongly oriented toward health promotion, resilience, and the creation of supportive living systems. Dietitians operate at the interface between food systems, human behavior, population health, and environmental sustainability, enabling action on upstream determinants rather than a narrow focus on clinical outcomes (6).

This viewpoint argues that while One Health depends on the engagement of all health professionals, dietitians play a distinct and influential role due to the systemic range of their practice. By shaping how food is produced, accessed, chosen, and consumed, dietitians contribute simultaneously to human health, animal welfare, and ecosystem resilience.

## One Health is a shared responsibility among health professionals

The operationalisation of One Health requires a fundamental reconceptualisation of professional roles and practice boundaries across the health sector. Rather than focusing exclusively on individual clinical outcomes, a One Health approach calls for collective and coordinated engagement with the structural determinants of health, including environmental, social, economic, and cultural factors. Addressing these determinants demands deliberate interprofessional collaboration, systems thinking, and shared accountability across professions (7).

Crucially, the interventions required to advance One Health are inherently cross-cutting rather than profession-specific. Health promotion, prevention, risk assessment, surveillance, behavior change support, continuity of care, and antimicrobial stewardship are shared domains of practice, enacted across professional roles and settings. Physicians, nurses, pharmacists, dietitians, and other health professionals contribute to common One Health objectives through complementary, overlapping, and mutually reinforcing actions.

A key challenge is that professionals themselves rarely recognize or explicitly frame these contributions within a One Health perspective. Many health professionals enact practices aligned with One Health principles without naming, conceptualizing, or evaluating them as such, reflecting the limited penetration of the paradigm within professional cultures (3). This phenomenon is not unique to contemporary practice. Historical analyses have shown that principles now associated with One Health were present in professional traditions long before the formal emergence of the concept, suggesting that alignment with One Health may precede explicit conceptual recognition (8).

Several factors contribute to this gap. Historically, One Health has been framed around zoonotic disease control and veterinary–environmental interfaces (9), narrowing its perceived relevance for many health professionals. In addition, One Health remains inconsistently integrated into undergraduate and postgraduate health professional education, limiting conceptual literacy, shared terminology, and interprofessional understanding (10). Health systems also tend to prioritize profession-specific competencies and biomedical outcomes, leaving little space for system-oriented and intersectoral perspectives. Empirical evidence supports this concern; for example, studies have shown that many physicians report limited or no awareness of One Health and minimal formal training exposure (10, 11).

A One Health perspective becomes operationally effective when health systems move beyond rigid role demarcations and foster coordinated interprofessional practice, in which responsibilities are shared, and expertise is reciprocally recognized (12). Such collaboration strengthens continuity across care pathways and alignment between individual care, community action, and population health strategies.

Within this interprofessional ecosystem, nutrition assumes a particularly integrative role. Its transversal scope, linking food systems, dietary behaviors, health promotion, chronic disease management, and environmental sustainability, positions nutritional practice as a connective thread across levels of intervention. Yet the same critical question applies: do dietitians explicitly perceive and articulate their work as contributing to One Health objectives? While direct empirical evidence is lacking, findings from other professional groups suggest that this awareness is likely limited. This conceptual invisibility simultaneously constrains implementation and represents a strategic opportunity to strengthen professional identity, education, and leadership within a One Health framework.

## Dietitians as key One Health actors in addressing contemporary health challenges

Non-communicable diseases represent one of the most significant global health challenges and are a central concern within a One Health perspective (2). Cardiovascular diseases, diabetes, cancer, digestive disorders, chronic respiratory diseases, and long-term neurological conditions share complex and interrelated determinants that extend far beyond individual biology. Dietary patterns, food environments, physical inactivity, environmental exposures, social inequalities, and ecosystem degradation all contribute to the development and progression of these conditions (13).

Beyond traditionally recognized chronic diseases, several contemporary nutrition-related challenges further illustrate the relevance of One Health. Obesity, food insecurity, unhealthy aging, and the growing consumption of ultra-processed foods emerge from complex interactions between food systems, social determinants, environmental conditions, and human behavior (14, 15). These challenges cannot be adequately understood through purely biomedical lenses, as agricultural practices, food environments, economic inequalities, climate-related disruptions, and broader ecological transformations shape them. From a One Health perspective, addressing these issues requires integrated strategies that simultaneously promote human well-being, sustainable food systems, and ecosystem resilience. Nutrition therefore becomes not only a determinant of health outcomes but also a strategic entry point for addressing interconnected challenges that affect people, animals, and ecosystems across multiple scales.

Within this context, dietitians play a critical role in translating One Health principles into actionable strategies for the prevention and management of non-communicable diseases. Their work addresses upstream determinants of health by shaping dietary patterns that support metabolic health, reduce chronic inflammation, and promote resilience throughout life. By advocating for diets rich in minimally processed foods, adequate in essential nutrients, and aligned with environmental sustainability, dietitians simultaneously contribute to individual health outcomes and reduce environmental pressures generated by unsustainable food systems (16, 17).

The increasing consumption of ultra-processed foods illustrates particularly well the interconnected nature of contemporary health challenges. These products have been associated with obesity, cardiovascular disease, diabetes, digestive disorders, certain cancers, and poorer mental health outcomes. At the same time, their production and distribution often depend on resource-intensive food systems with significant environmental impacts (18–20). Similarly, food insecurity remains closely linked to climate change, biodiversity loss, disruptions in agricultural productivity, and social inequalities, demonstrating how ecological and social vulnerabilities converge to influence nutritional health (21–23). This interconnectedness is perhaps most visible within the gastrointestinal system, which functions as a major interface between humans and their biological and biochemical environment. Food is not merely a source of nutrients; it also carries microorganisms, metabolites, environmental contaminants, and other exposures capable of influencing digestive and systemic health. In this sense, nutrition and digestive health represent a tangible expression of One Health, where human well-being is shaped by continuous interactions with wider ecological and biological systems (24). Consistent with this perspective, growing evidence indicates that dietary patterns influence gut microbiota composition and function, with important consequences for metabolic, immune, digestive, and neurological health (25, 26). The gut microbiota therefore exemplifies how health emerges from complex interactions extending beyond the individual organism and reinforces the systemic logic underpinning One Health.

Healthy aging further highlights the strategic importance of nutrition within this framework. As populations age, maintaining functional capacity, preventing frailty, preserving cognitive health, and supporting quality of life become increasingly important public health priorities (27, 28). Nutrition plays a central role in these processes, not only through the prevention and management of chronic diseases but also through its influence on resilience, independence, and long-term wellbeing. By addressing nutritional determinants across the life course, dietitians contribute to healthier aging trajectories while supporting the sustainability of health and social care systems.

Importantly, dietitians operate across multiple levels of intervention. At the individual level, they support people in adopting and sustaining dietary behaviors that reduce the risk of non-communicable diseases (16, 17). At the community and population levels, they contribute to health promotion programmes, school and workplace nutrition policies, and public health strategies that create supportive food environments (29–31). At the systems level, their involvement in food policy, regulation, and sustainability initiatives reinforces the alignment between non-communicable disease prevention and planetary health goals (32, 33).

Concrete examples further illustrate the leadership potential of dietitians within a One Health framework. In schools, dietitians contribute to the development of healthier food environments that promote lifelong healthy eating habits while encouraging more sustainable dietary choices. In communities facing food insecurity, they may coordinate or support nutrition programmes that improve access to healthy foods and address nutritional inequities linked to social and environmental determinants of health. Within nursing homes, day-care centers and healthy aging initiatives, dietitians play important roles in promoting nutritional adequacy, preventing malnutrition, and supporting the maintenance of health and quality of life across the life course. Increasingly, dietitians also contribute to policy discussions on sustainable diets, food labeling, and strategies to reduce the consumption of ultra-processed foods, helping align public health objectives with environmental sustainability goals. These examples demonstrate that dietitians are not merely participants in One Health initiatives but can also act as strategic leaders in developing and implementing integrated solutions that connect nutrition, population health, and sustainable food systems.

The relevance of nutrition within One Health extends beyond population-level interventions and policy development. It is equally evident in the management of complex long-term conditions, where biological, environmental, behavioral, and social determinants interact across the life course.

Long-term neurological conditions, such as Parkinson's disease, stroke, multiple sclerosis, dementia, and other chronic neurodegenerative disorders, illustrate particularly clearly the relevance of a One Health approach. These conditions are influenced by lifelong exposures, including nutrition, gut microbiota drivers, environmental toxins, social determinants, and lifestyle factors, and they often coexist with other non-communicable diseases (34–36).

Dietitians play a central role in supporting people living with prolonged neurological conditions by addressing both disease-related and system-level challenges. Adequate nutrition supports functional capacity, neuroplasticity, comorbidity management, and quality of life, and dietary interventions are increasingly recognized as relevant pathways influencing neurological disease trajectories and outcomes (37, 38).

From a One Health standpoint, dietitians also consider how environmental, social, and economic factors, such as food quality, contamination, access to healthy foods, and socio-economic vulnerability, shape the lived experience of people with neurological conditions. By working closely with other health professionals, dietitians contribute to integrated care models that support long-term self-management, adherence to rehabilitation programmes, and sustained health-promoting behaviors.

## Making One Health explicit in nutrition practice

If dietitians already contribute to One Health objectives through everyday practice, often without explicitly naming, framing, or evaluating their actions within this paradigm, the key challenge is how to make this contribution visible, intentional, and strategically strengthened. Advancing One Health, therefore, does not require creating new roles for dietitians, but rather the reframing, reinforcement, and amplification of existing practice.

The first essential step is strengthening conceptual awareness and literacy of One Health within the nutrition profession. Without a shared language or framework, contributions remain fragmented and undervalued. Explicitly positioning nutrition practice within a One Health paradigm enables dietitians to recognize their work as part of an integrated agenda linking human, animal, and ecosystem health, reinforcing professional identity and connecting routine interventions to systemic outcomes such as environmental sustainability, animal welfare, and population resilience.

Education is a critical leverage point. Integrating One Health principles into undergraduate and postgraduate nutrition curricula as cross-cutting lenses, rather than isolated modules, can normalize systems thinking, interprofessional collaboration, and sustainability-oriented decision-making. Continuing professional development further supports this process by linking nutrition interventions explicitly to One Health outcomes in practice-relevant ways.

Making nutrition's contribution intentional also requires structural inclusion of dietitians in interprofessional teams, policy forums, and One Health initiatives. In these contexts, dietitians can assume strategic leadership roles in dietary sustainability, food literacy, long-term condition self-management, and environmental risk mitigation, reinforcing their role as a bridging profession across clinical care, public health, and system-level action.

Finally, strengthening the dietitian's role within One Health depends on how interventions are evaluated, incentivised, and valued. Moving beyond short-term clinical indicators to include environmental and social co-benefits can make nutrition's One Health contribution more visible. Framing nutrition research explicitly within a One Health lens further legitimizes the profession as a core and indispensable contributor to integrated health agendas.

## Conclusion

One Health will remain an aspirational concept unless it is translated into everyday practice by health professionals and embedded within professional identities, education, and health system structures. Making One Health explicit in nutrition practice is not about expanding professional scope, but about recognizing, strengthening, and strategically positioning existing interventions within an integrated health paradigm. By embedding One Health into nutrition education, interprofessional collaboration, research, and evaluation frameworks, dietitians can move from implicit contributors to intentional leaders within One Health agendas. Strengthening this role is not optional; it is essential to addressing the interconnected challenges of chronic disease, environmental degradation, and health inequities in a sustainable, systems-oriented manner.

## Data Availability

The original contributions presented in the study are included in the article/supplementary material, further inquiries can be directed to the corresponding author.
